# Bicycle safety in Bogotá: A seven-year analysis of bicyclists’ collisions and fatalities

**DOI:** 10.1016/j.aap.2020.105596

**Published:** 2020-09

**Authors:** Germán A. Carvajal, Olga L. Sarmiento, Andrés L. Medaglia, Sergio Cabrales, Daniel A. Rodríguez, D. Alex Quistberg, Segundo López

**Affiliations:** a School of Economics, Universidad de los Andes, Bogotá, Colombia; b School of Medicine, Universidad de los Andes, Bogotá, Colombia; c Department of Industrial Engineering, Center for Optimization and Applied Probability, Universidad de los Andes, Bogotá, Colombia; d Department of City and Regional Planning, Institute for Transportation Studies, University of California, Berkeley, USA; e Urban Health Collaborative at the Dornsife School of Public Health, Drexel University, Philadelphia, USA; f Department of Environmental & Occupational Health, Dornsife School of Public Health, Drexel University, Philadelphia, USA; g Health and Road Safety Department, World Resources Institute Ross Center for Sustainable Cities, Bogotá, Colombia

**Keywords:** Bicycling mortality, Built-environment, Vision zero, Latin America

## Abstract

Road safety research in low- and middle-income countries is limited, even though ninety percent of global road traffic fatalities are concentrated in these locations. In Colombia, road traffic injuries are the second leading source of mortality by external causes and constitute a significant public health concern in the city of Bogotá. Bogotá is among the top 10 most bike-friendly cities in the world. However, bicyclists are one of the most vulnerable road-users in the city. Therefore, assessing the pattern of mortality and understanding the variables affecting the outcome of bicyclists’ collisions in Bogotá is crucial to guide policies aimed at improving safety conditions. This study aims to determine the spatiotemporal trends in fatal and nonfatal collision rates and to identify the individual and contextual factors associated with fatal outcomes. We use confidence intervals, geo-statistics, and generalized additive mixed models (GAMM) corrected for spatial correlation. The collisions’ records were taken from Bogotá’s Secretariat of Mobility, complemented with records provided by non-governmental organizations (NGO). Our findings indicate that from 2011 to 2017, the fatal bicycling collision rates per bicyclists’ population have remained constant for females while decreasing 53 % for males. Additionally, we identified high-risk areas located in the west, southwest, and southeast of the city, where the rate of occurrence of fatal events is higher than what occurs in other parts of the city. Finally, our results show associated risk factors that differ by sex. Overall, we find that fatal collisions are positively associated with factors including collisions with large vehicles, the absence of dedicated infrastructure, steep terrain, and nighttime occurrence. Our findings support policy-making and planning efforts to monitor, prioritize, and implement targeted interventions aimed at improving bicycling safety conditions while accounting for gender differences.

## Highlights

• Standardized bicycling collision rates have decreased in Bogotá in the last 7 years.
• Seven main geographic areas of bicycling risk were identified in Bogotá.
• Risk factors associated with bicycling mortality differ by sex.
• Findings support policy-making to implement targeted interventions to improve safety.
• Methodology based on open-data sources to permit replication and monitoring.

## 1 Introduction

Bicycling provides substantial benefits to the health and wellbeing of the population and is relevant for the development of a healthy and sustainable environment (Deenihan and Caulfield, 2014; Oja et al., 2011). Among children and adolescents, bicycling can prevent obesity and improves cardiorespiratory fitness (Oja et al., 2011; Sarmiento et al., 2015). Among adults, bicycling is associated with risk reduction for all-cause and cancer mortality; with risk reduction for cardiovascular, diabetes, cancer, and obesity morbidity (Celis-Morales et al., 2017); and with improvement in mental health (Mueller et al., 2015). Hence, bicycling may result in reductions in health care costs (Ding et al., 2016; Elvik, 2000) and increases in the gross domestic product (Fishman et al., 2015). Furthermore, increasing bicycling for commuting and recreation can mitigate greenhouse gas emissions, improve air quality, and can also reduce noise and vehicular congestion (Gössling et al., 2019; Krizec, 2007; Macmillan et al., 2014; Rabl and de Nazelle, 2012).

Despite the benefits of bicycling, research has shown that concerns related to traffic safety constitute a significant adoption barrier for bicycling (DiGioia et al., 2017). A significant percentage of the population across cities and countries are interested in bicycling but remain concerned about safety (Dill and McNeil, 2017; Pettit and Dogde, 2014). In this context, improving traffic safety for bicyclists is an essential goal of transportation and public health policies. Furthermore, the World Health Organization (WHO) has identified traffic collisions as the leading cause of death among children and young adults, mainly in low- and middle-income countries (Hazen and Ehiri, 2006; World Health Organization, 2018). Globally more than half of all road traffic deaths are among vulnerable road users, including bicyclists, pedestrians, and motorcyclists (World Health Organization, 2015).

In high-income countries, the proportion of bicyclists’ fatalities out of all fatal collisions is mostly less than 10 %, but it has been as high as 32 % in the Netherlands in the last decade (World Health Organization, 2013). However, mortality rates of bicyclists in these settings have decreased since 1990 (Buehler and Pucher, 2017; Valette, 2016). In Latin America and the Caribbean, the proportion of all road fatalities corresponding to bicyclists ranges from 0.5 % in the Dominican Republic and Ecuador and up to 12.7 % in Cuba (World Health Organization, 2015). The limited information on bicyclists´ collisions in Latin America shows a steady increase in the frequency of bicycle-related fatalities. For example, Mexico City (Mexico) (Secretaría de Salud de Los Estados. Unidos de México, 2016), Santiago de Chile (Chile) (Gobierno de Chile - Comisión Nacional de Seguridad de Tránsito, 2018; Gobierno de Chile, 2016), and Bogotá (Colombia) (Concejo de Bogotá, 2018; Gil et al., 2009), have reported an increase of the frequency of bicycle-related fatalities over the last decade. Specifically in Colombia, road crashes constitute a public health concern; in 2016, road traffic injuries were the second leading source of mortality by external causes (Ministerio de Salud y Protección Social, 2016).

Nevertheless, these reports in bicycle-related fatalities have substantial limitations. First, the frequency trends in mortality do not adjust for changes in exposure rates such as the number of trips, distance, or bicyclists’ population size. Second, these reports do not show variation in bicycling fatalities by age-group or sex, despite qualitative evidence showing important differences (Buehler and Pucher, 2017; Lin et al., 2017). Finally, these reports fail to identify intracity variations in bicyclists’ fatality risk; nonetheless, this information is crucial to prioritize policies and programs aimed at preventing collisions and fatalities among bicyclists (Yiannakoulias et al., 2012).

Currently, there is a body of research focused on high-income countries aimed at understanding how individual and contextual factors impact the severity of bicyclists’ collisions (DiGioia et al., 2017). However, ninety percent of road traffic fatalities are concentrated in low- and middle-income countries (Watkins and Sridhar, 2009; Welle et al., 2018), but research in these locations is limited, including the Latin American region. This highly urbanized region has been characterized by innovative, inclusive, and sustainable transportation policies that have the potential to promote safe bicycling (Diez Roux et al., 2018; Quistberg et al., 2018). Thus, there is both an urgent need and an opportunity to improve Latin American urban policy-making in bicyclist safety based on data analysis and sound evidence (Bliss, 2004).

Salient among large Latin American cities, Bogotá (Colombia) has developed a comprehensive policy framework to support safe bicycling. These policies have a multi-sectoral perspective, including the mobility, sports and recreation, education, and health sectors. There are five main policies and programs to promote bicycling in Bogota. The first is the national policy *Ley Probici* (Bicycle Law) which gives both a half-day free from work and a free trip in public transport for every 30 bicycle-trips to work; the law also makes mandatory to have parking spaces for bicycles in all car parking lots (Congreso de Colombia, 2016). The second program is the local *Plan Bici of Bogota* (Bicycle plan for Bogotá) which main objective is to make bicycling the primary mode of transport for the citizens of the city through its infrastructure, safety, culture, environment, and health components (Secretaría Distrital de Movilidad, 2016). The third program is *Al Colegio en Bici* (Let’s bike to school) which is part of the city’s school transport strategy to eliminate the access barriers to education for low-income students by providing bicycles and safe routes to children living 2–3 km away from school (Hidalgo et al., 2016). The fourth program is the *Ciclovía* of Bogotá (Open Street program on Bogotá) in which every Sunday and holidays, during 7 h, the main streets are closed to motor vehicles and open exclusively to individuals for bicycling, walking, running, and other leisure activities (Torres et al., 2013). The last policy is the global road traffic safety policy known as *Vision Zero* (Sweden Government 1996/97:137 et al., 2020Sweden Government 1996/97:137 et al., 2020; Welle et al., 2018) to which Bogotá committed to in 2016. The bicycling policies implemented in Bogotá have the potential of increasing the number of bicyclists (Ahmad et al., 2001; Hoehner et al., 2008; Verma et al., 2015), but evidence regarding bicyclists safety is limited.

In this context, in the current study, we examine spatiotemporal trends and potential contextual risk factors explaining bicyclists’ collisions and fatalities in Bogotá (Colombia) for the 2011–2017 period. Specifically, the aims of the study are: (i) to analyze temporal trends in bicyclists mortality and non-fatal collision rates standardized by the bicyclists’ population, and per- vehicle kilometers traveled (VKmT); (ii) to identify areas of high bicyclists’ mortality; and (iii) to determine the individual and contextual risk factors associated with bicyclists’ mortality.

## 2 Literature review

Bicycling safety is an active research field recently enriched with multiple perspectives provided by different data analysis techniques (DiGioia et al., 2017; Reynolds et al., 2009; Vecino-Ortiz et al., 2018). Studies assessing bicycling safety differences over time have used multiple methods, including the evolution of risk approaches in Seville (Spain) (Marqués and Hernández-Herrador, 2017), longitudinal deaths analysis in the United States (Cummings et al., 2006), rates per capita comparisons in Organization for Economic Co-operation and Development (OECD) member countries (Buehler and Pucher, 2017), and assessments of the impact of policy on risk and exposure (Mooney et al., 2018). The findings in these studies indicate improvements in safety conditions when controlling for exposure variables and standardizing bicycle collision counts per bicycle trips or capita. The *safety in numbers* hypothesis (Jacobsen et al., 2015; Jacobsen, 2003) is one of the explanations for this relationship between greater exposure and reduced relative risk. These results underscore the importance of introducing exposure variables when studying bicycling safety risk overtime to support policies aiming to increase the number of bicyclists, such as the provision of dedicated infrastructure (Cervero et al., 2009; Dill and McNeil, 2017) and promotion strategies including social media and incentives (Bagloee et al., 2016; Fishman et al., 2015; Martens, 2007).

Geospatial analysis has also been applied to bicyclists’ safety research (Chen et al., 2017) to identify collision-prone hotspots. Lawrence et al. (2015) used geospatial analysis to explore trends in bicyclists’ collisions in Melbourne (Australia). They found that a combination of behavior and road infrastructure changes contributed to a reduction of collisions in a limited section of the city with dedicated bike lanes. Yiannakoulias et al. (2012) propose a methodology for mapping a measure of bicycling risk in Hamilton (Canada) using a standardized rate of reported collisions per VKmT within confined areas. Their results showed intracity variations in bicycling safety that could target future infrastructure investments. Minikel (2012) compared collision rates and collisions’ severity on arterial roads and bicycle boulevards in Berkeley (US), concluding that boulevards (i.e., wide streets, usually lined with trees) are significantly safer environments for bicyclists. In Latin America, researchers have conducted geographical location studies about all-road-users’ and pedestrians’ collisions but have not studied bicyclists. Fox et al. (2015) investigated the risk distribution of pedestrian fatalities in Cali (Colombia), identifying high-priority hotspot-areas for policy prevention strategies in the city. Rodríguez et al. (2003) investigated the location of all traffic collisions in Bogotá (Colombia), finding that they concentrate along main corridors and intersections.

A variety of other methodological approaches have been used to examine bicyclist safety over time and space, and its associations with contextual and individual risk factors. Econometric models are widely used to study associations to derive policy recommendations (Chen et al., 2017; Lin et al., 2017; Prabhakar and Rixey, 2017). Other methodologies include Impact Evaluation assessment (Jensen, 2007; Marqués and Hernández-Herrador, 2017), Latent Class Clustering (Kaplan and Prato, 2013), among many others (DiGioia et al., 2017; Reynolds et al., 2009).

Accordingly, prior research has shown that contextual characteristics, including built and social environments such as road, traffic, land use, and time of day, are associated with changes in bicyclist safety. For example, roads with improved street lighting and suitable pavement conditions show a lower risk of fatal bicyclists’ collisions (Moore et al., 2011; Reynolds et al., 2009). Moreover, wide roads, high employment density, and increased land-use mixture are also associated with reduced fatal bicyclists’ collision risk (Chen et al., 2017; Chen and Shen, 2016). On the contrary, rural environments (Kaplan and Prato, 2013), high levels of traffic flow (Zhang et al., 2012), nighttime cycling (Chen et al., 2017), roads with poor network design and connectivity (Johnson et al., 2010), presence of large vehicles (Moore et al., 2011), and industrial land use (Reynolds et al., 2009) have a higher risk of bicyclist’ fatalities.

Studies examining other contextual variables have shown mixed results. These contextual variables include intersections (see Chen and Shen, 2016 and Moore et al., 2011), speed limits (see Kaplan and Prato, 2013 and Lawrence et al., 2015), and signaling (Chen et al., 2017; Johnson et al., 2010). Nonetheless, the most relevant variables with mixed results are dedicated bicycle infrastructure (see Jensen, 2007 and Marqués and Hernández-Herrador, 2017), and *Level of Traffic Stress* (LTS) measurements (Mekuria et al., 2012), since these are both critical policy planning and intervention tools (Dill and Carr, 2003; Macmillan et al., 2014).

Individual characteristics are also significant explanatory variables associated with the bicyclists’ safety. Studies in high-income countries show that females, children, and the elderly have the highest risk of experiencing a fatal bicyclists’ collision (Garrard et al., 2008; Lin et al., 2017). Safety devices, such as reflective clothing and bicycle helmets, have been associated with lower severity outcomes (Chen and Shen, 2016) and demonstrated to prevent specific types of head injuries (DC et al., 2010; Høye, 2018; Olivier and Creighton, 2017). However, evaluations of mandatory laws show mixed results in mortality reduction (Vecino-Ortiz et al., 2018). Also, inattentive driving and the use of alcohol and drugs are associated with more severe bicyclists’ collisions (Ballestas and Flórez Valero, 2011; Johnson et al., 2010; Kaplan and Prato, 2013).

Despite the diversity of modeling approaches, there is a lack of consensus of a single theoretical framework to relate risk factors and outcomes to deliver general policy recommendations, in particular those applicable to the global south. Studies show that factors vary across geographies, frequently leading to mixed results. Therefore, there is a need to develop evidence with data from the global south that shares similar characteristics. We believe this is the first investigation in Colombia focused on bicycling safety. Having the particular characteristics of a middle-income country in Latin America, the methodology and the findings of this investigation contribute to filling the knowledge gap on the effectiveness of injury prevention interventions among one of the most vulnerable transportation modes (Vecino-Ortiz et al., 2018).

## 3 Data and study site

### 3.1 Study site

Bogotá, the capital of Colombia, has a population of 7.18 million inhabitants (Departamento Administrativo Nacional de Estadística, 2018), who make 17.2 million trips every day (Transconsult and Infometrika, 2015a). The city has an average temperature of 14 °C with alternate periods of rain and drought rather than seasons. Bogotá is considered to be among the top 10 most bike-friendly cities in the world (Elisabeth Yorck von Wartenburg, 2019; Sagaris, 2015). In 2015, Bogotá had the largest network of dedicated bicycle pathways in Latin America with over 479 km, including the following types of bicycle infrastructure: (i) off-street bicycle lanes located in sidewalks, verges and central medians (*ciclorutas*); (ii) segregated on-road bicycle lanes (*ciclobandas*); (iii) paint-delimited on-road bicycle lanes (*banda ciclopreferente*); (iv) shared-use bicycle lanes (*carril bus-bici*); (v) and traffic-calming lanes (Ministerio de Transporte de Colombia, 2016). Additionally, during the Sunday’s *Ciclovía* program the city temporarily adds 126 km to the bicycle network by making a selection of main streets open exclusively for people so that they can enjoy safe and free space for cycling, skating, walking, and jogging, among other activities (Instituto Distrital de Recreación y Deporte et al., 2020). Despite the dedicated infrastructure and worldwide recognition, the modal share of bicycle trips in Bogotá is less than 5 %, according to the 2015 mobility survey (Transconsult and Infometrika, 2015a).

### 3.2 Collisions

Our collisions data sources include: (i) the official yearly local traffic collisions data reported by Bogotá’s Secretariat of Mobility (for years 2011–2015); and (ii) collisions data compiled by the Fundación Despacio and World Resources Institute (for years 2013–2017) from the Bogotá’s Secretariat of Mobility. Both sources include all collisions reported to the police occurring in public spaces, including roads and parks, among other areas. Collisions that occurred in private property are not reported to the police. We use multiple sources to reduce underreporting (Kaplan and Prato, 2013) related to changes in the city government staff. After cleaning the data for duplicate registries using the date, time, location, gender, and outcome of the collision, we identified 366,814 unique collisions of all types among all road users during the seven-year period. We georeferenced all the unique fatal and nonfatal collisions using the coordinates recorded in the data sources, if available, and the recorded addresses through the Google Maps geocoding Application Programming Interface (API) (Google, 2020). The final dataset resulted in 10,043 geocoded bicyclists’ collisions, including single-bicycle collisions and collisions with motor vehicles or other bicyclists, with one observation per each bicyclist involved per collision. We removed the collisions with individuals under five-years-old due to missing values on sociodemographic and exposure to risk indicators. The final sample included 9950 bicyclists’ collisions, including 358 fatal events reported for the period 2011–2017 in the city of Bogotá.

#### 3.2.1 Fatal and nonfatal collisions

In Bogotá, reported nonfatal collisions are included in official records by the police at the crash site. Agencies only record official fatalities after a calendar year is complete. In February, annually, the National Forensics Agency (Instituto Nacional de Medicina Legal y Ciencias Forenses) and the Secretariat of Mobility compare the collected data about collisions’ severity. Police officers classify the collisions’ severity at the crash site into six categories using a local scale (including levels: [i] property damage only; [ii] not-injured; [iii] injured; [iv] injured with no hospital care; [v] injured with hospital care; and [vi] dead). Nonetheless, severity categorization is not consistent across years due to procedural changes (Secretariat of Mobility, personal meeting, May 24th of 2018). As a result, we only use the fatal/nonfatal classification in our analyses, as fatal events are subject to stricter supervision and reporting, which makes its categorization consistent across years.

### 3.3 Exposure to risk indicators for collisions

#### 3.3.1 Bicyclists’ population

We estimated the bicyclists’ population from Bogotá’s household travel surveys (in Spanish *Encuestas de Movilidad*) of 2005, 2011, and 2015; and from the multipurpose household survey (in Spanish *Encuesta Multipropósito*) of 2014 and 2017 taking into consideration the sample design representative weights for users. All of the five surveys have been conducted by Bogotá’s local government (Dirección de Metodología y Producción Estadística - DIMPE - Dirección Técnica and Departamento Administrativo Nacional de Estadistica - DANE, 2014; Dirección de Metodología y Producción Estadística - DIMPE - Dirección Técnica Departamento Administrativo Nacional de Estadistica - DANE, 2017; Secretaría de Transito y. Transporte and Alcaldia Mayor de Bogotá D.C., 2005; Transconsult and Infometrika, 2015b; Unión Temporal Steer Davies and Gleave - Centro Nacional de Consultoría, 2011). The number of bicyclists is restricted to the commuter population defined as “a person who lives in Bogotá and uses the bicycle as the main transportation mode for utilitarian purposes on a daily basis” (e.g., trips to and from sites of work and school, among others). We use this subset of the population because it entails travel to and from high-utility, obligatory activities and is therefore useful for understanding people´s typical travel behaviors (Verma et al., 2015).

#### 3.3.2 Vehicle kilometers traveled

We calculated daily VKmT using the reported origins and destinations (OD) from Bogotá’s travel surveys (2005, 2011, and 2015) using the sample design representative weights for trips (Secretaría de Transito y Transporte and Alcaldia Mayor de Bogotá D.C., 2005; Transconsult and Infometrika, 2015b; Unión Temporal Steer Davies and Gleave - Centro Nacional de Consultoría, 2011). The surveys register the OD of all trips in the city using 849 small areas called Transportation Administration Zones (ZATs, Zonas Administrativas de Transporte). We estimated the routes and distances between ZATs using the shortest-path by bicycle between ZAT centroids using Open Source Routing Machine (OSRM) (Luxen and Vetter, 2011). Considering the ZATs span - on average - 2 km^2^ within the city, we did not consider randomized origins and destinations (Yiannakoulias et al., 2012). Though we only have data on collisions between 2011 and 2017, we used all the data available for the estimation of exposure to risk indicators, including the 2005 travel survey. We decided to include 2005 data since it was the only travel survey with actual travel distances allowing us to assess the performance of our estimates. We evaluated our estimations by comparing the actual and estimated daily VKmT (sum of distances multiplied by the number of trips reported in the survey). When stratifying by sex, age by quinquennial groups, and OD by *Localidad* (Bogota’s largest administrative districts), the average error over the 732 stratified groups was 5.7 %. The error of the total daily VKmT without stratification was 3.4 % (actual daily VKmT = 1,100,707 km; estimated daily VKmT = 1,063,762 km).

#### 3.3.3 Linear interpolation for the missing years

Since there are available surveys in five points in time, to fill the estimates of bicyclists’ population and the daily number of trips over the years considered for our analyses, we used (i) stratified linear interpolations between each pair of available years and (ii) a linear extrapolation with the last two available years when estimating the number of trips after 2015. Missing years for the estimates of the bicyclists’ population include 2006–2010, 2012–2013, and 2016. Missing years for the daily number of trips include 2006–2010, 2012–2014, and 2016−2017. We stratified by sex, age by quinquennial groups, reported residence location by *Localidad* for the population, and OD by *Localidad* for the bicycle trips.

### 3.4 Independent variables

Independent variables include individual characteristics, contextual attributes at the point of occurrence of the collision, and variables of the Urban Planning Zone (UPZ, Unidad de Planeamiento Zonal) unit at which the collision takes place. UPZ divisions are the official administrative planning and data reporting zones of Bogotá’s public administration (Alcaldía Mayor de Bogotá, 2004). There are 117 UPZs in Bogotá, so they are larger on average than the ZATs.

In addition to the collision event, the police register some individual, contextual, and environmental characteristics at the crash site. These data include bicyclists characteristics (sex, age, driver’s license, bicycle age), other crash-involved vehicle types (TransMilenio, cargo vehicle, bus, automobile, motorcycle, and other), and space-time and environmental features (weekday, time of occurrence, weather conditions, and infrastructure design) as shown in Appendix Table 1. We use the bicycles’ age as a proxy of the riders’ experience and bicycle familiarity (as an approximation of the time they have been riding).

For the built-environment variables, we rely on secondary data sources. We compute the terrain slope from the Japan Aerospace Exploration Agency (JAXA) global digital surface model (Tadono et al., 2014) at the crash site according to the georeferenced coordinates. We also consider characteristics measured at the UPZ, including information related to land use (area; entropy index; and commercial, residential, office, and commercial share), built environment (public space area; lighting and crowdedness conditions according to the SAFETIPIN scale [Secretaría Distrital de Movilidad, 2016]; and count of road surface failures), bicycle infrastructure (pathway km; traffic signals and lights), and economic characteristics (assessed land value in USD/m^2^) as shown in Appendix Table 2. We collected these data from multiple geographical information system (GIS) sources including (i) Bogota’s geospatial data agency (IDECA, Infraestructura de Datos Espaciales para el Distrito Capital) (Concejo de Bogotá, 2004), (ii) the Bogota’s bureau of land management (UAECD, Unidad Administrativa Especial de Catastro Distrital) (Concejo de Bogotá, 2006), (iii) Bogota’s urban development institute (IDU, Instituto de Desarollo Urbano) (El Concejo del Distrito Especial de Bogotá, 1972), (iv) Bogota’s bureau of public services (UAESP, Unidad Administrativa Especial de Servicios Publicos) (Alcaldía Mayor de Bogotá, 2012), and (v) the national bureau of statistics (DANE, Departamento Administrativo Nacional de Estadística) (Presidencia de la República de Colombia, 1951). We sampled these contextual characteristics at a yearly frequency (at year’s end). For variables with missing yearly data, including road surface failures, lighting and crowdedness conditions, we imputed the value from the last recorded year.

Finally, we include the input variables that allow us to compute the LTS for road segments and intersections (Huertas et al., 2020) at the crash site. These measurements (input variables) comprise lane width, average motor vehicles’ speed, average congestion, vehicles’ density, average vehicles’ flow, number of lanes, the presence/absence of bicycle infrastructure, and the presence/absence of public transportation system (SITP, Sistema Integrado de Transporte Público) routes (a proxy for the presence of heavy vehicles). To estimate the values of the average motor vehicles’ speed, average congestion, vehicles’ density, and the average vehicles’ flow, we retrieved the average and free-flow transit times for every road segment using Google’s distance matrix API. We use the traversing time along the segment at 24:00 of Tuesday, September 4, 2018, as a proxy for the free-flow traversing time (Huertas et al., 2020). We included the LTS rating from 1 to 4, as shown in Appendix Table 3, and also considered only differentiating LTS level 4 (higher stress = 1) from all other levels (levels 1, 2, and 3 = 0).

The exposure to risk indicators measures are also aggregated at the UPZ level. Bicyclists’ population is defined by those who reported their home location within the UPZ according to the surveys. Daily VKmT are estimated with the georeferenced shortest paths with as follows. First, using the UPZ base map, the paths are divided into shorter segments, where each segment is fully contained within a UPZ. Second, for each segment, we measure the length (VKmT of one trip) and multiply by the number of trips on the segment to obtain an estimate of the daily VKmT on that particular shortest path (from which the segment was derived) within the UPZ. Finally, we add the daily VKmT on all segments contained within the UPZ to compute the total daily VKmT at the UPZ level. A graphical explanation of the procedure is shown in Appendix Fig. 1.

## 4 Methodology

### 4.1 Fatal and nonfatal trend analysis

We conducted a trend analysis stratified by sex of the standardized fatal and nonfatal collision-counts during the seven-year period from 2011 to 2017. First, we calculated the direct standardized monthly collision rates based on: (i) the total bicyclists’ population; and (ii) VKmT. Second, using the 12 monthly rates for each year, we calculated the average monthly rates and 95 % confidence intervals (95 % CI). We also applied indirect standardization to estimate the standardized mortality ratio (SMR) per month (Curtin and Klein, 1995) using the World Health Organization World Standard Population 2000–2025 (Ahmad et al., 2001). We computed age-specific-death-rates separately for men and women by quinquennial age groups (Ministerio de Salud y Protección Social, 2016) and compared bicycle-specific death trends with the behavior exhibited by all the fatal collisions in the city of Bogotá from 2011 to 2016. As with direct standardization, using the 12 monthly rates for each year, we calculated the average monthly SMR and 95 % CI.

We used multiple standardization methods to enable comparisons during the sampling period against other municipalities (Jensen, 2007; Minikel, 2012; Reynolds et al., 2009; Valette, 2016; Vecino-Ortiz et al., 2018). Additionally, by using exposure metrics, we can reflect a scale-independent measure of bicycling risk, avoiding interpretation errors associated with other changes (e.g., population, number of bicyclists) that can create unnecessary confusion in the media and the general population (Concejo de Bogotá, 2018).

### 4.2 Fatal and nonfatal point-pattern and distribution analyses

We conducted point-pattern and distribution analyses of the events to identify high-risk geographical areas for bicyclists’ fatalities and nonfatal collisions. First, we conducted a concentration pattern analysis over the separated groups of fatal and nonfatal collisions estimating Ripley’s K function over the points to detect geographical clustering in the location of the collisions (Baddeley, 2008). The Ripley’s K function is a tool for analyzing mapped spatial process data by estimating a relationship between the number of events and the density of the events; it can be used to unveil relationships between the stochastic pattern and its clustering properties (Dixon, 2002). The identification of a clustered pattern within collisions sharing the same outcome may indicate an association of the severity with the location and its characteristics. Second, we used a quadrat count dispersion test analysis to statistically test for differences in the rate of occurrence of fatal events by area (collisions per km^2^) (Diggle, 2013; Howroyd, 1996). In this analysis, the study region is divided into rectangles (called ‘quadrats’) of equal size, and the number of points in each rectangle is counted. The rate of events by area has well-defined hypothesis testing, allowing us to detect zones with a high concentration of fatal collisions. We used the area as a denominator in this analysis since the exposure to risk indicators would be zero in many regions of the city, limiting the analysis. We selected the grid size for the quadrat count using the 95 % confidence contour values for the fixed-bandwidth isotropic Gaussian kernel estimate of the point process (Diggle and Fisher, 1985). A fixed-bandwidth isotropic Gaussian kernel corresponds to a Gaussian mixture of homoscedastic distributions centered on each point in space. We used the kernel contour (isoline at 95 % cumulative probability) to avoid bias and subjectivity in the selection of the grid size. To prevent overfitting of the observed pattern, we calibrated the Gaussian kernel variance parameter using leave-one-out cross-validation to maximize the point process likelihood (Baddeley and Turner, 2005; Moore, 2001). We define the high-risk areas as those in the top 5 % of the rate of the number of fatal collisions per km^2^.

### 4.3 Generalized additive mixed model

To determine the magnitude and association of the individual and contextual factors with bicyclists’ fatalities (among all collisions), we fit a generalized additive mixed model (GAMM) with bicyclists involved at collisions as the unit of analysis. A generalized additive model (GAM) is a semi-parametric model with linear predictors involving a sum of smooth functions of covariance (Hastie and Tibshirani, 1990; Wood, 2013). We used standard thin plate regression splines as the smooth functions for all continuous variables, with a fast restricted maximum likelihood (fREML) parameter estimation method (Wood, 2006). The GAM has been proven to be more flexible and often outperforms the Generalized Linear Model (GLM) since the GAM does not impose restrictions on monotonic relationships (Zhang et al., 2012). To account for the nested structure of the indicators within the UPZ and the repeated cross-sectional datasets, we use GAMM to (i) relax the independent and identically distributed (IID) condition (Moore et al., 2011); (ii) allow unobserved heterogeneity across collisions accumulated over time, which is relevant in transportation safety analyses (Mannering et al., 2016); and (iii) control for the unobserved time-effects. We assume that the number of fatalities follows a binomial distribution. The GAMM allows us to quantify the association between the probability of death of a bicyclist during a collision and the individual and contextual variables (Fahrmeir and Lang, 2001; Powers, 2012). We included the coordinates of the events as smoothing terms to account for the possible existence of spatial correlation (Chen and Shen, 2016). We included the exposure to risk indicators as regressors in the model to control for the intensity of bicycling within the UPZ.

We used three steps in the GAMM estimation. First, we selected the covariates to be included as those with a bivariate significance smaller than 0.2 (Bursac et al., 2008), with a missing count less than 10 %, and with no significant collinearity between them. Second, we conducted a multivariate analysis with complete cases exploring heterogeneous effects for females and males separately. Third, given the class imbalance problem of fatal and nonfatal collisions, we conducted a random over-sampling as a sensitivity analysis (Abd Elrahman and Abraham, 2013; Larose, 2006). All the analyses include robustness checks such as (i) training the model with the balanced dataset; and (ii) estimating an additional set of models including the variables that exceeded 10 % of missing values but met the significance criteria simultaneously with interactions between the time of occurrence and other relevant factors. All statistical analyses were conducted in R (R Development Core Team, 2011) using the MGCV package for fitting the GAMM model (Wood, 2013).

The ethics committee of the School of Economics at Universidad de los Andes approved the protocol of the study (Facultad de Economía, 2019).

## 5 Analysis and results

### 5.1 Trends of fatalities and collision rates

**Fig. 1:**
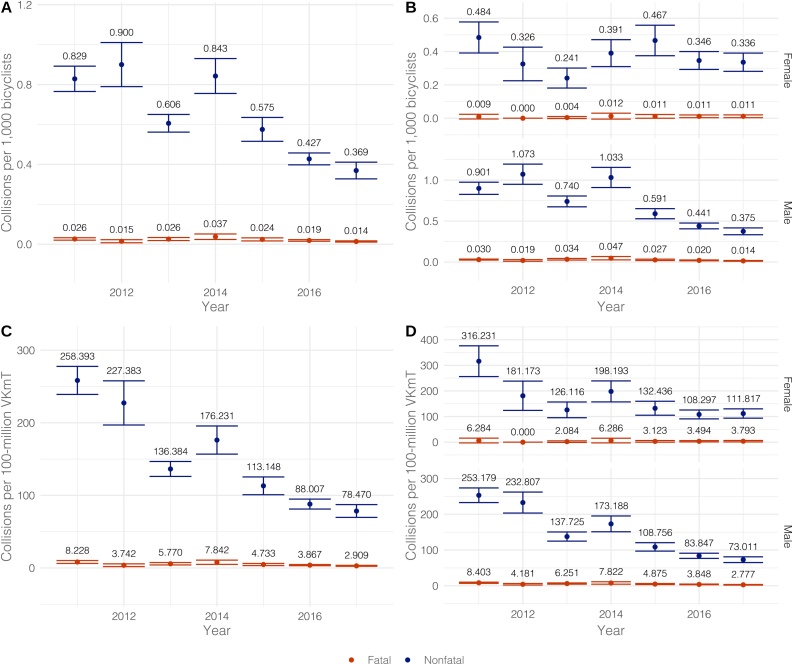
Yearly averages of monthly standardized rates of fatal and nonfatal bicyclists’ collisions (points) with 95 % confidence interval (error bars), and reported mean estimates for the city of Bogotá for the period 2011 to 2017 with different denominators: *A.* estimated number of bicycle users. *B.* estimated number of bicycle users by sex. *C.* estimated traveled kilometers. *D.* estimated traveled kilometers by sex.

The analysis (Fig. 1) shows an overall decrease in nonfatal collision rates between 2011–2017, independent of the exposure to risk indicator. For the overall population, the standardization by total bicyclists’ population shows a reduction of 55 % (p < 0.001) between the years 2011 and 2017. However, the magnitude of the reduction differs by sex (p < 0.001), with a magnitude of 30 % (p = 0.007) for females, and 58 % (p < 0.001) for males. For the overall population, the standardization by VKmT shows a reduction of 70 % (p < 0.001) between the years 2011 and 2017, and there are no significant differences by sex in the reduction (p = 0.426).

Fatal collision rates also present statistically significant decrements between 2011 and 2017, for both when standardizing by the bicyclists’ population and VKmT. For the overall population, the standardization by total bicyclists’ population shows a reduction of 46 % (p = 0.001) between the years 2011 and 2017. However, the magnitude of the change differs by sex (p = 0.037), with no change (p = 0.796) for females, and a decrease of 53 % (p < 0.001) for males. For the overall population, the standardization by VKmT shows a reduction of 64 % (p < 0.001) between the years 2011 and 2017, and there are no significant differences by sex in the reduction (p = 0.491). Furthermore, during the period 2011–2016 for the overall bicyclists’ population, the monthly average SMR shows a reduction from 3.81 to 2.11 (45 % [p < 0.001]) that does not show differences by sex (p = 0.745). This reduction provides additional evidence for the findings with the standardized collision rates.

### 5.2 Geographical areas with high-risk of fatal collisions

**Fig. 2:**
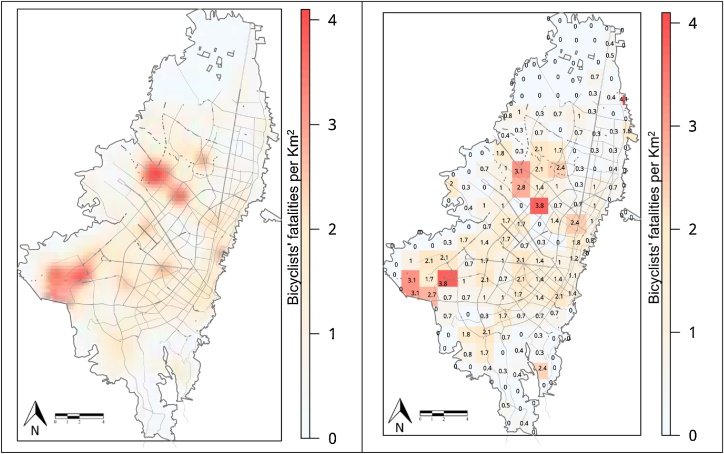
Bicyclists’ mortality in Bogotá for the period 2011 to 2017. Fixed-bandwidth kernel estimate with an isotropic Gaussian kernel (left) and quadrat count density approximation with fatal collisions rate per square kilometer (right). Hotter-colored (red) areas indicate high-risk locations. Low-income zones: (i) Casablanca in the Kennedy Localidad – 3.8 fatalities/km^2^, (ii) León XII in the Bosa Localidad – 3.1 fatalities/km^2^, and (iii) Santa Rita in the San Cristobal Localidad – 2.4 fatalities/km^2^). Middle-income zones: (iv) Calle 68 with Avenida Boyacá – 3.8 fatalities/km^2^, (v) San Felipe in the Barrios Unidos Localidad – 2.4 fatalities/km^2^, and (vi) Autopista Medellín with Avenida Ciudad de Cali – 3.1 fatalities/km^2^. High-income zone: (vii) Calle 127 with Avenida Boyacá – 2.5 fatalities/km^2^ (For interpretation of the references to colour in this figure legend, the reader is referred to the web version of this article).

The results of the concentration pattern analysis shown in Fig. 2, reveal that there is a shorter distance between similar severity collisions than what would be observable in the absence of spatial correlation or clustering. The outcome dependence on the contextual risk factors is one possible explanation for the clustering pattern of the collisions grouped by severity. The Gaussian kernel on the left shows the estimated probability distribution of the collisions highlighting the clustered structure. Using the quadrat analysis, we created delimited areas for study, enabling us to compare same-size areas and detect specific zones where the rate per km^2^ of fatal bicyclists’ collisions is statistically higher than those from their surroundings. We identified seven higher-risk locations across the city. Among these areas, three are located in low-income zones, three areas correspond to middle-income zones, and one high-risk area is located in a high-income zone.

### 5.3 Factors associated with bicyclists’ fatal collisions

Table 1 includes the estimated linear coefficients for the GAMM models for females (column 1), males (column 2), whole population (column 3), and the robustness exercise with the balanced sample (column 4). The results show that fatal collisions are associated with the vehicle type involved in the collision. For females, a fatal collision is more likely when colliding with a cargo vehicle, buses, and other vehicle types compared to an automobile. For males, a fatal collision is less likely when colliding with a motorcycle compared to an automobile. Overall, a fatal collision is more likely when colliding with a vehicle more voluminous than an automobile (cargo vehicles, TransMilenio, buses, and other vehicle types). The nonsignificant coefficients show patterns in the model that become significant with the balanced sample, like those of weekday, bicycle pathway, LTS level 4, and the presence of SITP.

**Table 1:** Predictors with linear effects in the baseline and additional GAMM specifications. Estimated marginal contribution to the log-odds ratio of a fatal bicyclist’s collision in Bogotá for the period 2011 to 2017.

Variables (Log-odds of a fatal bicyclist's collision)	(1) Females	(2) Males	(3) Whole population	(4) Blanced sample^b^
Linear terms				
Sex (1 = Male)			0.064	0.025
			(0.202)	(0.101)
Vehicle type§ (baseline = Automobile)				
TransMilenioª	−14.552^c^	1.889 ***	1.197 ***	2.476 ***
	(8,597.970)	(0.488)	(0.484)	(0.311)
Cargo vehicle	3.765 **	2.189 ***	2.271 ***	2.840 ***
	(1.184)	(0.245)	(0.236)	(0.149)
Bus	2.481 *	0.949 ***	1.051 ***	1.455 ***
	(1.126)	(0.228)	(0.219)	(0.116)
Motorcycle	0.622	−0.756 *	−0.687 *	−0.845 ***
	(1.473)	(0.350)	(0.337)	(0.152)
Other^d^	2.251 *	0.543 **	0.650 **	0.982 ***
	(1.115)	(0.211)	(0.205)	(0.131)
Weekday (baseline = Sunday)				
Monday	−0.731	−0.272	−0.311	−0.228
	(0.866)	(0.299)	(0.280)	(0.147)
Tuesday	−0.748	0.186	0.102	0.338 *
	(0.861)	(0.271)	(0.256)	(0.142)
Wednesday	−0.111	0.024	0.006	0.183
	(0.817)	(0.277)	(0.263)	(0.140)
Thursday	−0.016	0.346	0.326	0.625 ***
	(0.820)	(0.264)	(0.249)	(0.140)
Friday	−0.556	−0.123	−0.192	−0.021
	(0.909)	(0.286)	(0.271)	(0.146)
Saturday	−0.353	0.262	0.199	0.502
	(0.936)	(0.268)	(0.255)	(0.141)
Bicycle pathway (1 = Police reported collision on a bicycle pathway)	−1.861	−0.442	−0.581	−1.608 ***
	(3.310)	(0.702)	(0.682)	(0.380)
SITP†† (1 = Presence of heavy vehicles)	2.245	0.659	0.823	1.719 ***
	(3.341)	(0.709)	(0.689)	(0.386)
LTS (1 = LTS 4)	−1.783	−0.481	−0.609	−1.607 ***
	(3.332)	(0.722)	(0.701)	(0.391)
Constant	−6.177 ***	−4.221 ***	−4.363 ***	−3.809 ***
	(1.235)	(0.291)	(0.332)	(0.225)
Random Intercept (std. Dev.)				
UPZ variation	0.002	0.001	0.001	0.454
Year nested in UPZ variation	0.962	0.421	0.457	2.390
Observations	1,120	6,896	8,016	11,031

Standard errors in parenthes is *** p<0.001, ** p<0.01, * p<0.05, · p<0.1. Note: Collisions of bicyclists less than 5 years old were removed for data availability limitations.

§ Largest vehicle related to the collision in the following preciding order: TransMilenio, Cargo vehicle, Bus, Motorcycle, and Other.

ª TranMilenio is Bogotá City's BRT system.

d Other includes: horse-drawn, train, ambulance, agricultural and industrial machinery.

†† SITP is Bogotá city's integrated public bus system other than BRT.

b Balanced using over-sampling of the minority class with a 30 % positive class target.

c There are only 4 registries of female bicyclists collisions with BRT vehicles.

**Fig. 3:**
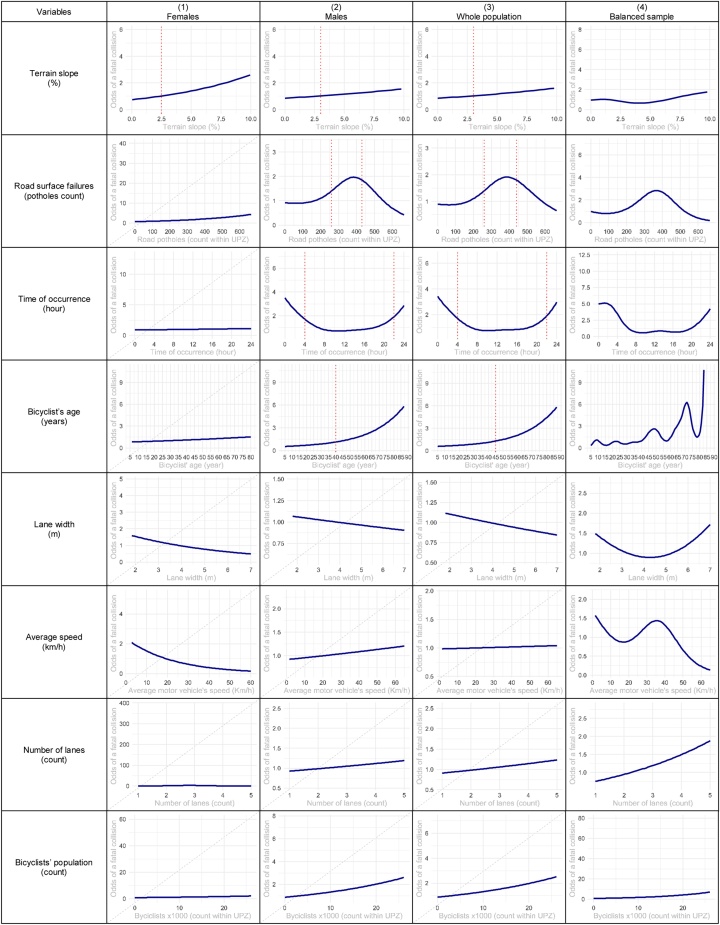
Predictors with nonlinear effects in the baseline and additional GAMM specifications. Estimated contribution to the odds ratio and 95 % confidence region of a fatal bicyclist’s collision in Bogotá for the period 2011 to 2017. Vertical dotted lines correspond to thresholds when the odds ratio becomes significantly different from 1. Strikethrough variables were not significant at a 5% significance level.

Fig. 3 includes the estimated nonlinear functions for the fitted GAMM models. For females, fatal collisions are positively associated with terrain slope with a threshold of 2.5 %. For males, fatal collisions are associated positively with the terrain slope with a threshold of 3%, road surface failures, the time of the occurrence, and the bicyclists’ age. Overall, the significant nonlinear predictors for fatal collisions include terrain slope, road surface failures, time of occurrence, and bicyclist’s age. For males and the whole population, the road surface failures show a bell-shaped curve indicating that moderate to poor road surface conditions are positively associated with a fatal collision. For males and the whole population, the time of the occurrence shows a U-shaped curve with the time of occurrence, indicating that collisions between 22:00 and 4:00 hours are more likely to be fatal. The model trained with the balanced sample shows additional significant associated factors, including lane width, average speed, number of lanes, and the bicyclists’ population count.

#### 5.3.1 Sensitivity analysis and subsamples

The robustness exercise with the balanced sample model shows consistency of the estimated coefficients and fitted nonlinear functions. The estimations retain unchanged the sign of the associations and modify the magnitude of the coefficients slightly. The significance of the relationships and the functional forms of the associations with lane width, average speed, and proportion of UPZ area used as office space, change due to the increase of the sample size but do not alter the general results.

We estimated additional models to assess infrastructure design, weather conditions, bicycle age, and the interactions of the time with the location and the lighting conditions, which are variables with more than 10 % of missing values. The additional models show that neither infrastructure design (separating segments and intersections [n = 5311]), nor weather conditions (n = 3603) are associated with the outcome of the collision. Nonetheless, bicycle age (a *proxy* of the bicyclists’ experience) is associated negatively with the odds of a fatal outcome (n = 2927). Regarding the interaction terms (n = 11,031), first, we found that a fatal collision is more likely to occur in the southeast of the city between 16:00 and 8:00; second, for females, we found that areas with lighting conditions worse than 40 % are positively associated with a fatal collision between 20:00 and 1:00.

## 6 Conclusions

We analyzed bicyclists’ collisions in Bogotá for the period 2011–2017. We showed that, during this period, bicycling fatal and nonfatal collision rates standardized by total bicyclists' population have decreased by 55 % and 46 %, respectively. This reduction differs by sex, indicating a larger reduction among men. Factors associated with collisions also differ by sex. Additionally, we showed that similar severity collisions exhibit a geographically-clustered pattern with high-risk areas located in the west, southwest, and southeast of the city.

The reductions in the overall collision rates could be explained, in part, due to the advocacy groups’ efforts, and policies and programs that have promoted safe bicycling in Bogotá. Policies and programs have differed between government periods (Rosas-Satizabal and Rodriguez-Valencia, 2019). Between 2004 and 2012, the city governments’ priorities were limited in terms of promoting bicycling. The few kilometers of bicycle pathways built during this period were already included in previous city plans and were not properly designed to improve network connectivity. From 2012 to 2017, the government capitalized on the multiple advocacy groups’ efforts, developing policies and programs including *Al Colegio en Bici* and the *Plan Bici* of Bogotá. We hypothesized that this context could have contributed to the three-fold increase in the population of bicyclists between 2011 and 2017, while collisions grew by 40 % over the same period. The decrease in mortality rates is also consistent with the safety in numbers hypothesis (Jacobsen, 2003) and with the findings by Buehler and Pucher (2017) in high-income countries in which fatality rates have decreased since 1990. Findings from studies focusing in high-income countries have shown reductions of the collision rates standardized by population ranging between 30 % and 68 % over 24 years, while our results show a reduction of 55 % over a shorter period of seven years. One explanation, in part, for this difference is that in 2009 the fatality rates per 100 million VKmT for the US (4.7) and Germany (1.3) (Buehler and Pucher, 2017) were much lower than the rate for Bogotá for 2011 (8.2). Importantly, when comparing the fatality rates of bicyclists with the average for all the road users in Bogotá, our findings indicate that the bicyclists’ population in the city presents a collisions’ fatality rate 111 % higher than what is observed for all road users.

Regarding the contextual factors associated with fatal collisions, we found that for women and men, colliding with a cargo vehicle, buses, and other vehicle types larger than an automobile, a time of occurrence between 20:00 and 4:00, and a terrain slope over 3% were positively associated with fatal collisions. Among females, we found that poor lighting conditions, and a terrain slope over 2.5 % are positively associated with a fatal outcome. Among males, we found that bicyclists over 40-years-old and traveling on moderate to poor road surface conditions are factors positively associated with fatal collisions. Factors negatively associated with fatal collisions among women and men included the presence of dedicated bicycle pathways, a lane width between 3.5 and 4.5 m, a motor vehicles’ average speed between 15 and 25 km/h, streets with less than three lanes, high LTS (level 4), and areas with an office space use greater than 7 %.

Our results regarding sex, age, vehicle type, and lighting conditions are consistent with previous research conducted in OECD countries (Chen et al., 2017; Garrard et al., 2008; Lin et al., 2017; Moore et al., 2011; Rodríguez et al., 2003). We found a similar pattern with the negative associations of increased employment density (Chen and Shen, 2016), and suitable pavement conditions (Reynolds et al., 2009) with fatal collisions. Importantly, we also found a pattern that indicates that the presence of bicycle pathways is negatively associated with fatal collisions, which is consistent with what has been reported for high income-countries (Marqués and Hernández-Herrador, 2017). Contrary to what we hypothesized, the LTS was negatively associated with fatal collisions. One possible explanation could be related to what was found by Chen et al. (2017), where the authors hypothesized that LTS 4 streets discourage less experienced bicyclists and attract highly skilled riders that may be able to avoid more severe injuries. That hypothesis may explain why lane width, average speed, and the number of lanes, show nonlinear associations with the probability of a fatal outcome.

Other factors commonly evaluated in the literature, including weather and infrastructure design, were found nonsignificant in Bogotá (DiGioia et al., 2017; Moore et al., 2011). Regarding weather conditions, one explanation could be the fact that Bogotá does not have seasons and has subtropical weather with two rainy periods. Regarding the differentiation between segments and intersections, one explanation for the nonsignificant association can be the lower variability in the motor vehicles’ speed due to congestion that does not differ at crossings.

Additionally, in the context of a city with relevant differences in elevation (from 2504 m to 3337 m), we found a new association between a steeper terrain slope and a more severe outcome in a collision. Second, we identified a significant association between less experienced bicyclists and an increased fatal collision probability. Third, we identified a potential cyclical term within bicycling risk associated with the weekday of occurrence. However, further research is needed on further background on bicyclists' experience and more resolution on intra-year changes of exposure indicators.

The intracity variations of bicycling risk identified are in line with previous findings in Canada (Yiannakoulias et al., 2012), and provides relevant information for prioritizing interventions aimed at reducing fatal and nonfatal collisions. Our risk areas are characterized by minimal to complete absence of bicycle infrastructure. Furthermore, when present, the bicycle pathways are located on wide streets that correspond to main corridors with a significant presence of cargo vehicles, TransMilenio (bus rapid transit system), buses, and other large vehicles. Additionally, there is limited connectivity and lack of direct paths on the network that forces the bicyclists to interact with motor vehicles and pedestrians, increasing the probability of collisions.

### 6.1 Limitations

The study has the following limitations. First, our data is subject to underreporting of collisions, but we could not assess its magnitude. The underreporting is expected to be more notable for the less severe collisions (i.e., [i] property damages only and [ii] not-injured) since these are more commonly not reported by individuals. Hence, our data might present bias towards more severe collisions. Second, while we used multiple data sources, it was not possible to link them to assess the accuracy of the source or to link to other sources. However, it is the only comprehensive available source of data for Bogotá to make inferences about the risk factors associated with bicycling. In order to report missing data and assess the accuracy of the registries, we would have needed ambulance records, medical records, data from the National Forensics Agency, and a survey of the collisions not reported by individuals, which is recommended for future studies. Third, we did not have information regarding the bicyclists’ driving direction at the collision (e.g., uphill or downhill). Fourth, considering all exposure to risk indicator values are only available by year, it was not possible to evaluate smaller time intervals or intra-year variations in exposure indicators (i.e., changes in the volume of trips per month). Fifth, missing years of the exposure to risk indicators were filled using interpolation and extrapolation; thus, there are no fit metrics for the trend estimates. Finally, collision reports do not differentiate among the bicycle infrastructure types; hence, we included the variable as presence/absence of any of the five infrastructure types present in the city’s network.

### 6.2 Policy recommendations

Our results have significant policy implications. The findings reported herein could inform policies like the New Urban Agenda and the United Nation’s Agenda for Sustainable Development in which understanding safe sustainable transport like bicycling is relevant to achieve the SDG 11 of making cities inclusive, safe, resilient, and sustainable by 2030 (United Nations, 2018).

Our results also provide evidence that supports national and local policies aimed at promoting safe bicycling in Bogotá and Colombia. The Bogotá’s Secretariat of Mobility could use our results to monitor, prioritize, and plan targeted interventions aimed at improving bicycling safety conditions. Specifically, we first recommend continuing the implementation of safe bicycle infrastructure, including dedicated bicycle pathways and separating bicyclists from environments with the presence of large vehicles. Second, we recommend continuing with programs like *Vision Zero* and law enforcement of reduced speed limits. Third, we identified priority geographical areas that could be intervened in the city. Lastly, our empirical results revealed gender-inequalities on mortality rates reduction and the factors associated with fatal collisions. This evidence could be used by the local government to design a public policy guideline with a gender and equality focus (Alcaldía Mayor de Bogotá, 2010; Peñalosa, et al., 2017). Specifically, the associated individual and contextual risk factors, suggest that safe bicycling campaigns should target females and newcomers using a public policy population approach (Peñalosa, et al., 2017), aiming interventions to affect factors that significantly differ from those that could affect male bicyclists. The joint programs from Bogotá’s Planning Secretariat, Secretariat of Women, and the Secretariat of Mobility could help increase safe bicycling among women. These programs currently support women bicycling advocacy groups and detect critically unsafe points through the SAFETIPIN program (Pinzón-Medina, 2018; Viswanath and Basu, 2015).

Additionally, since we propose a methodology entirely based on multiple open-data sources, it allows the replication of the analyses potentially serving as a tool for monitoring safety conditions and evaluating the impact of the policy interventions to measure the effect of coordinated interventions. Moreover, our results and methodology could be applied in other cities from Latin America and the global south.

## Financial disclosure

The study was partially funded by The Salud Urbana en América Latina (SALURBAL) / Urban Health in Latin America project funded by the Wellcome Trust [205177/Z/16/Z]. More information about the project can be found at www.lacurbanhealth.org. Also, the project was partially supported by the Research Office at Universidad de Los Andes through the seed grant “Finalización de Proyectos” of 2018. Segundo López received funding from World Resources Institute through the Bloomberg Initiative for Global Road Safety, Bogota, Colombia. This research did not receive any specific grant from funding agencies in the public, commercial, or not-for-profit sectors. The funding sources had no role in the analysis, writing, or decision to submit the manuscript.

## CRediT authorship contribution statement

**Germán A. Carvajal:** Conceptualization, Resources, Project administration, Methodology, Formal analysis, Investigation, Writing - original draft, Writing - review & editing, Visualization. **Olga L. Sarmiento:** Conceptualization, Methodology, Formal analysis, Resources, Writing - review & editing, Supervision, Project administration, Funding acquisition. **Andrés L. Medaglia:** Conceptualization, Methodology, Resources, Writing - review & editing, Supervision, Project administration, Funding acquisition. **Sergio Cabrales:** Conceptualization, Methodology, Formal analysis, Writing - review & editing. **Daniel A. Rodríguez:** Methodology, Validation, Writing - review & editing. **D. Alex Quistberg:** Methodology, Validation, Writing - review & editing. **Segundo López:** Methodology, Validation, Writing - review & editing, Data curation.

## Declaration of Competing Interest

None.
